# The association between altered intestinal microbiome, impaired systemic and ocular surface immunity, and impaired wound healing response after corneal alkaline-chemical injury in diabetic mice

**DOI:** 10.3389/fimmu.2023.1063069

**Published:** 2023-01-31

**Authors:** Yashan Bu, Kendrick Co Shih, Ho Lam Wong, Sum Sum Kwok, Amy Cheuk-Yin Lo, Joseph Yau-Kei Chan, Alex Lap-Ki Ng, Tommy Chung-Yan Chan, Vishal Jhanji, Louis Tong

**Affiliations:** ^1^ Department of Ophthalmology, Li Ka Shing Faculty of Medicine, University of Hong Kong, Pokfulam, Hong Kong SAR, China; ^2^ Department of Ophthalmology, Hong Kong Sanatorium and Hospital, Hong Kong, Hong Kong SAR, China; ^3^ Department Ophthalmology, University of Pittsburgh Medical Center, Pittsburgh, PA, United States; ^4^ Cornea and External Eye Disease Service, Singapore National Eye Centre, Singapore, Singapore; ^5^ Ocular Surface Research Group, Singapore Eye Research Institute, Singapore, Singapore

**Keywords:** intestinal microbiome, diabetes, corneal wound healing, alkaline chemical injury, T-cell mediated immunity, ocular surface

## Abstract

**Purpose:**

We aim to investigate the effect of sustained hyperglycemia on corneal epithelial wound healing, ocular surface and systemic immune response, and microbiome indices in diabetic mice compared to controls after alkaline chemical injury of the eye.

**Methods:**

Corneal alkaline injury was induced in the right eye of Ins2^Akita^ (Akita) mice and wild-type mice. The groups were observed at baseline and subsequently days 0, 3, and 7 after injury. Corneal re-epithelialization was observed under slit lamp with fluorescein staining using a cobalt blue light filter. Enucleated cornea specimens were compared at baseline and after injury for changes in cornea thickness under hematoxylin and eosin staining. Tear cytokine and growth factor levels were measured using protein microarray assay and compared between groups and time points. Flow cytometry was conducted on peripheral blood and ocular surface samples to determine CD3+CD4+ cell count. Fecal samples were collected, and gut microbiota composition and diversity pattern were measured using shotgun sequencing.

**Results:**

Akita mice had significantly delayed corneal wound healing compared to controls. This was associated with a reduction in tear levels of vascular endothelial growth factor A, angiopoietin 2, and insulin growth factor 1 on days 0, 3, and 7 after injury. Furthermore, there was a distinct lack of upregulation of peripheral blood and ocular surface CD3+CD4+ cell counts in response to injury in Akita mice compared to controls. This was associated with a reduction in intestinal microbiome diversity indices in Akita mice compared to controls after injury. Specifically, there was a lower abundance of Firmicutes bacterium M10-2 in Akita mice compared to controls after injury.

**Conclusion:**

In diabetic mice, impaired cornea wound healing was associated with an inability to mount systemic and local immune response to ocular chemical injury. Baseline and post-injury differences in intestinal microbial diversity and abundance patterns between diabetic mice and controls may potentially play a role in this altered response.

## 1 Introduction

Diabetes mellitus (DM) is a significant health problem worldwide. It is associated with sight-threatening ocular complications and represents the most common causes of blindness in working age populations. Apart from retinal diseases, corneal disease is another major complication of DM, affecting up to 70% of all diabetic patients (1, 2). Clinically, diabetic keratopathy is characterized by an impaired cornea epithelial wound healing response, which leads to higher risks of recurrent cornea erosion syndrome, infectious keratitis, sterile corneal ulcers, and ultimately corneal blindness due to scarring. The clinical manifestations of diabetic keratopathy can be explained by the detrimental effect of sustained hyperglycemia on the cornea, resulting in reduced secretion of growth factors on the ocular surface during corneal injury, damaged corneal sub-basal nerves with loss of reduced levels of neurotrophic factor, and increased apoptosis of cornea epithelial cell (3, 4). Despite major leaps in our understanding of the pathogenesis of diabetic keratopathy in the past decade, there remains a lack of therapeutic strategies in clinical practice. While published *in vivo* and *in vitro* studies have examined the use of topical therapeutic agents, including substance P (5) and aloe vera (6), in diabetic cornea disorders, there is still a need to explore glucose-independent systemic therapies that may have ameliorated long-term complications of diabetes across multiple body systems.

With the advent of next-generation deep sequencing in metagenomics over the last two decades, we now understand the importance of gut microbiome in the development and programming of host metabolism and immunity (7). From new evidence gathered, the composition of our gut microbiome plays an important role in the development of autoimmune diseases, including demyelinating disease, inflammatory bowel disease (IBD), and autoimmune uveitis (8). The gut microbiome reportedly acts a key mediator in balancing the immune response between the anti-inflammatory regulatory T (Treg) cells and the pro-inflammatory helper T (Th) 17 cells at the mucosal surface (9–13). Recent interest has been focused on the metabolic effects of gut commensals, particularly in their role in the development of obesity, insulin resistance, and metabolic syndrome. Researchers discovered that high-fat diets were associated with particular detrimental patterns of intestinal microbiota, termed dysbiosis (14). This conferred a pro-metabolic syndrome state to the host, who subsequently developed obese and insulin-resistant clinical phenotypes. These metabolic effects could be transferred to healthy lean individuals through fecal transplantation techniques to give rise to a similar pro-insulin resistant state. Thus, the intestinal microbiome may be an important lever for manipulation in the management of metabolic syndrome and type 2 diabetes. Considering the potential of the gut microbiome to alter both host metabolism and immunity concurrently, it is important to point out that diabetic patients have been shown to have altered intestinal T-cell immunity, potentially as a result of endotoxemia (15). Thus, the development of microvascular complications, e.g., diabetic keratopathy, and the altered immune response in diabetic patients may be potentially correlated with the altered gut microbiome composition in diabetics.

To further investigate this relationship between gut microbiome, immunity, and complications in diabetics, we employed a diabetic cornea wound healing model in rodents. We used a controlled alkaline burn injury of consistent concentration, exposure time, and surface area on the corneas of heterozygous Akita mice. Wild-type mice were used as controls. Chemical injury was selected as a method of insult as it creates a cornea epithelial wound and induces an inflammatory response on the ocular surface, thereby allowing us to investigate both corneal epithelial wound healing and ocular surface immunity (16–18).

## 2 Methodology

### 2.1 Breeding and selection of heterozygous Akita mice

Mice heterozygous for the Akita spontaneous mutation (Ins2^Akita^) were used in experiments. This is a model of type I diabetes, with heterozygous Akita mice developing hyperglycemia, hypo-insulinemia, polydipsia, and polyuria starting at 3–4 weeks of age (19). Wild-type (WT) female and heterozygous Akita male mice aged 8–12 weeks were used for mouse breeding. The animals were kept in a temperature-controlled animal room subjected to a 12-h light/12-h dark cycle provided with water supply and sufficient food. All the animal care and experimental procedures conformed to the Association of Research in Vision and Ophthalmology (ARVO) Statement for the Use of Animals in Ophthalmic and Vision Research and were approved by the Committee on the Use of Live Animals in Teaching and Research (CULATR, 4696-18) of the University of Hong Kong. Blood glucose was initially measured when the mice reached 5 weeks of age to determine whether the offspring phenotype was Akita or WT.

### 2.2 Blood glucose measurement

Sufficient amount of blood sample (~5 μl) was collected from the saphenous vein. Briefly, hair around the saphenous vein was removed and a puncture is made to obtain a sufficient blood sample with a capillary tube. A blood glucose test strip together with a blood glucose meter (Contour plus) was used to conduct the measurement. Hemostasis was achieved by applying gentle pressure with a sterile cotton bud to the puncture wound. Blood glucose was measured at 5 weeks after birth in order to identify Akita mice and WT mice, and was further determined at day 0, day 3, and day 7 after alkaline injury of the cornea, as previously described (19).

### 2.3 Induction of corneal alkaline injury

To induce chemical injury, an alkaline burn was conducted on the mouse cornea. For general anesthesia, the animal will receive intraperitoneal injection of ketamine (0.6 mg/100 μl/10 g body wt) and xylazine (0.15 mg/100 μl/10 g body wt). Local analgesic was applied on the mouse cornea prior to the injury. A 1.5-mm-diameter filter paper was soaked with 0.1 M NaOH and covered the center of the cornea for 10 s. Then, the cornea and conjunctival sac were rinsed with filtered water for 30 s immediately. Antibiotics were applied to avoid infection. Corneal re-epithelialization was examined under a slit lamp with fluorescein stain at day 0, day 3, and day 7 after the injury, as previously described (20).

### 2.4 Hematoxylin and eosin staining

The excised corneas were immediately fixed in 4% paraformaldehyde in phosphate-buffered saline (PBS) overnight. The corneas were then dehydrated with increased gradient of ethanol and finally with chloroform overnight. After that, the corneas were infiltrated with paraffin wax [Tissue PrepTM Embedding Media (Certified), Fisher Chemical] and embedded using the Shandon Histocentre2 Embedding Station, Midwest. Sagittal corneal sections with 5 μm thickness were prepared using a microtome (HM 315 Microtome, Microm). Sections were mounted on the positively charged microscope slides (Lab’IN Co) and dried. Corneal sections were deparaffinized and stained with hematoxylin for 30 s and eosin for 5 s. The sections were dehydrated and mounted with DPX mountant (06522, Sigma-Aldrich). Only central corneal sections were chosen for measuring corneal thickness, and the average of the two measurements was used for analysis, as previously described (20).

### 2.5 Tear protein microarray assay

Mouse tear samples were collected by placing a Schirmer’s strip at the mouse conjunctiva for 5 min. The wet part of the strip was cut off and the length was measured. The surface area of the cut-out pieces and the respective tear volume were calculated on ImageJ. The total protein concentration was measured with a Nanodrop. Total protein concentration was used as a normalization value and calculated all data points per total protein and generated Prism graphs (21). An unpaired *t*-test was conducted to compare the tear protein levels between WT and Akita samples, as well as between time points.

### 2.6 Flow cytometry

Peripheral blood was collected from the mouse tail vein with a restrainer. Blood (150 to 200 μl) was collected in 1.5-ml Eppendorf tubes containing EDTA. Red blood cells were lysed with Red Blood Cell lysis buffer and centrifuged at 250*g* for 5 min. Ocular surface samples were excised from the mice and were digested in collagenase at 37°C overnight. The cells were filtered with a 100-μm cell strainer. Then, the lymphocyte cells or ocular surface cell samples were washed three times with flow cytometry buffer (Invitrogen) and were incubated with rat anti-mouse CD4 antibody attached to FITC fluorophore and anti-mouse CD3 antibody (Invitrogen) attached to PE for 1 h. Then, the cells were washed three times with flow cytometry buffer for and then the samples were subjected to flow cytometry immediately. The flow cytometry was conducted with BD FACSCanto II (HKU core facility). Compensation was calculated by single-stain sample with FITC and PE. A CD3+CD4+ T-cell prevalence was obtained by FlowJo software for the diabetic and WT samples, as previously described (22).

### 2.7 Shotgun sequencing of intestinal microbiome

Mouse fecal samples were collected at baseline, day 3, and day 7 by putting the mice individually in single cages for 30 min. DNA samples were extracted, and microbiota species present in the sample were identified by shotgun sequencing followed by bioinformatic analysis using statistical and computational methods (23). Raw, unassembled reads were used as input and matched the sequence against reference databases of known bacteria, viruses, fungi, protists, and antibiotic resistance genes. Microbiota diversity pattern was presented by heat map and alpha diversity index.

### 2.8 Genotyping with polymerase chain reaction gel electrophoresis

Tail tips (1-3cm in length) were collected after the mice were sacrificed. The tips were cut into tiny pieces (approximately 2 mm in length). The DNA samples from each mouse was extracted from the samples, and polymerase chain reaction (PCR) was conducted for DNA amplification and measurement (19).

### 2.9 Statistical analysis

The data were presented as mean and standard deviation. A Pearson chi-squared test was used to compare differences in percentage of diabetic and WT mice healed at day 3 and day 7. An unpaired *t*-test was used to compare differences in blood glucose level, protein level in the tear sample, and alpha diversity pattern of intestinal microbiome and between WT and Akita mice; a paired *t*-test was performed to compare changes in tear protein concentration between baseline level and day 0, day 3, and day 7 after the injury. One-way ANOVA was used to analyze longitudinal changes of microbiota composition and tear protein level. When *p* < 0.05, the samples were considered significantly different. All statistical analyses were conducted using GraphPad Prism 9 (California, USA). Additionally, the gut microbiota diversity pattern was analyzed using the alpha diversity indices, Chao1, Simpson, and Shannon.

## 3 Results

### 3.1 Blood glucose measurement and genotyping of WT and Akita mice

**Figure 1:**
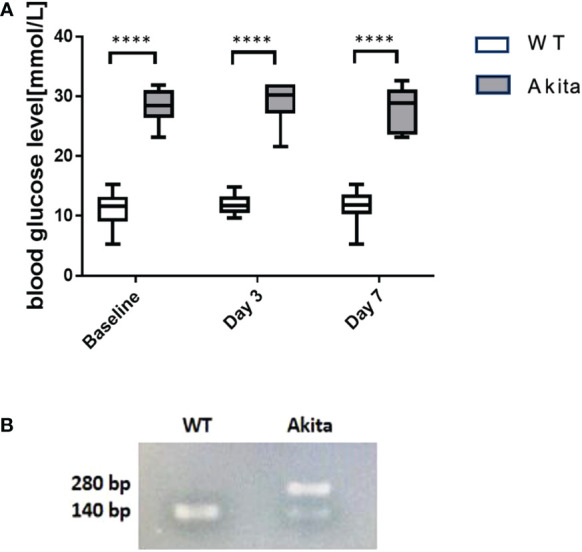
Result of blood glucose level monitoring of WT and Akita mice. **(A)** Result of blood glucose level monitoring of WT and Akita mice. **(A)** Blood glucose measurement at baseline, day 3, and day 7. Unpaired *t*-test shows that Akita mice have significantly higher blood glucose level as compared to WT mice at baseline (before injury), day 3, and day 7 after injury. **(B)** Confirmation of WT and Akita mice by genotyping with PCR gel electrophoresis;, two DNA strands of WT mice are both able to be digested by enzyme Fnu4HI while heterozygous Akita mice have one mutated DNA strand that cannot be digested; thus, one band is present in the lane containing WT mice DNA sample whereas two bands present in that containing Akita mice DNA samples (*n* = 50 in WT, *n* = 50 in Akita). ****p<0.0001.

Blood glucose was measured before induction of the corneal alkaline burn injury, at day 3 and day 7 after the injury, respectively. Compared to WT mice at baseline, Akita mice had a significantly higher blood glucose level (27.95 ± 1.446 mmol/L) compared to WT mice (11.42 ± 0.4621 mmol/L) at baseline as well as at day 3 (28.95 ± 1.522 mmol/L vs. 11.17 ± 1.477 mmol/L) and day 7 (28.09 ± 0.6987 mmol/L vs. 11.71 ± 0.3653 mmol/L) after injury (**Figure 1A**) (*p* < 0.001, *n* = 55). Additionally, genotyping was conducted with the genomic DNA of WT and Akita mice to confirm the presence of the mutated insulin 2 gene (*Ins2*) (**Figure 1B**).

### 3.2 Significantly delayed cornea epithelial wound healing after corneal alkaline injury in Akita mice compared to WT mice

**Figure 2:**
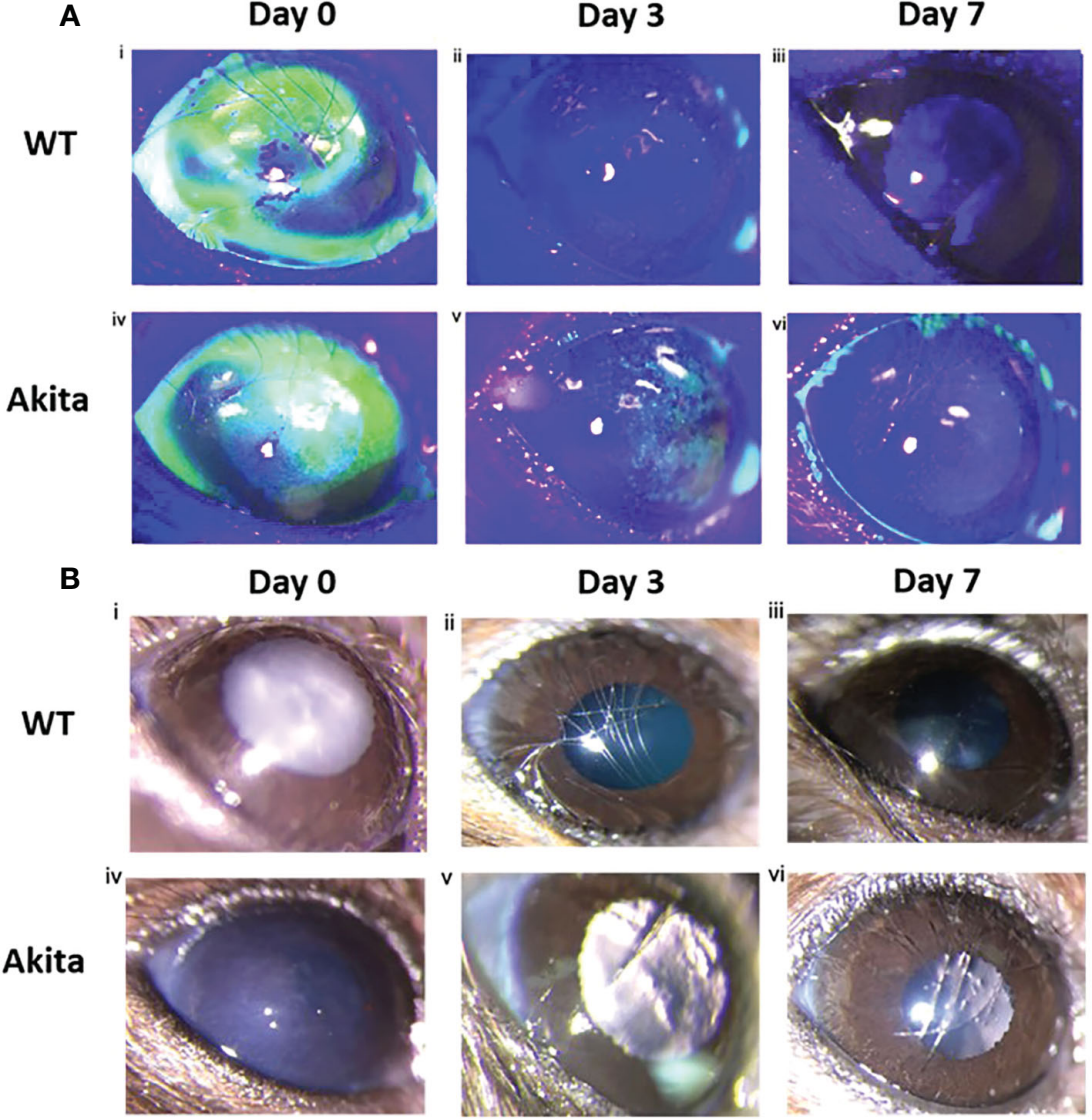
Wound healing analysis for WT and Akita mice with slit lamp images. **(A)** Injured cornea from WT and Akita mice at days 0, 3, and 7 after injury using 0.1 M NaOH and 10 s injury time, with fluorescein stain under cobalt blue light. Photos of the corneas taken immediately after injury showed significantly impaired corneal epithelium for both WT and Akita mice. (i, iv) The cornea from WT mouse was fully re-epithelized at day 3 after injury (ii); the cornea from Akita mouse was not fully re-epithelized at day 3 (v) and day 7 (vi) after injury. Magnification:40×. **(B)** Bright-field images of the right (injured) cornea from WT and Akita mice taken at day 0 (i, iv), day 3 (ii, v), and day 7 (iii, vi) after corneal alkaline injury (*n* = 55 in WT, *n* = 55 in Akita).

Differences in degree of re-epithelialization between Akita mice and WT mice were compared with the help of fluorescein staining and examination under cobalt blue filter immediately after injury (day 0) as well as on day 3 and day 7 after injury. Fluorescein dye stained the de-epithelialized cornea surface. The representative photos of slit lamp examination showed that alkaline burn with 0.1 M NaOH and 10 s injury time induced significant cornea epithelial injury as indicated by the presence of green fluorescein under cobalt blue light (**Figure 2A-i, iv**) and the cornea opacity in the bright-field images (**Figure 2B-i, iv**). The presence of green fluorescein under cobalt blue light for Akita cornea was clearly visible till day 3 after injury (**Figure 2A-v**), whereas for WT mice, the green fluorescein was not present at day 3 after injury (**Figure 2A-ii**). The presence of corneal opacification was visible from the bright-field images for the cornea from Akita mouse (**Figure 2B-v**), whereas no opacification was visible since day 3 after injury for WT mice (**Figure 2B-ii, iii**).

**Figure 3:**
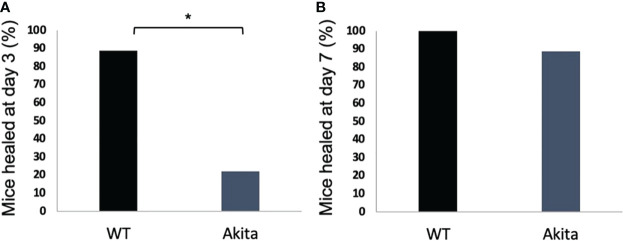
Percentage of diabetic and WT mice healed at day 3 after the corneal alkaline injury. **(A)** Pearson chi-squared test was performed (confidence level = 95% and p =0.0044, *n = 9*) and indicated that a significantly greater percentage of WT mice were healed than diabetic mice at day 3. **(B)** Pearson chi-squared test was performed and indicated no significant difference between the percentage of WT mice and diabetic mice healed at day 7 (p > 0.9999, *n = 9* for both the WT and Akita groups). *p<0.05.

A chi-square test was performed to determine an association between the healing rate of corneal epithelium and the presence of sustained hyperglycemia. Contingency tables for the number and percentage of WT and Akita mice healed at day 3 and day 7, respectively, are as shown in **Tables 1** and **2**. Results of the chi-square test analysis demonstrated that Akita mice exhibited significantly delayed wound healing compared to WT mice after corneal alkaline burn injury; i.e., at day 3 after the corneal alkali injury, significantly more WT mice (88.9%) had achieved complete corneal re-epithelialization than Akita mice (22.2%) (*n* = 9, *p* = 0.0044) (**Figure 3A**; **Table 1**), proving that diabetes significantly impaired cornea wound healing.

**Table 1:** Contingency table for Pearson chi-square test analysis of number and percentage (in brackets) of WT and Akita mice healed at day 3 after corneal alkaline injury (degrees of freedom = 1, significance level = 0.05).

		Healed	Not Healed	Total
**Akita**	Observed number	2(22.2%)	7(77.8%)	9
	Expected number	4.5(50%)	4.5(50%)	
**WT**	Observed number	8(88.9%)	1(11.1%)	9
	Expected number	4.5(50%)	4.5(50%)	
**Total**		9(100%)	9(100%)	18

**Table 2:** Contingency table for Pearson chi-square test analysis of number and percentage (in brackets) of WT and Akita mice healed at day 7 after corneal alkaline injury (degrees of freedom = 1, significance level = 0.05).

		Healed	Not Healed	Total
**Akita**	Observed number	8(88.9%)	1(11.1%)	9
	Expected number	4.5(50%)	4.5(50%)	
**WT**	Observed number	9(100%)	0	9
	Expected number	4.5(50%)	4.5(50%)	
**Total**		9	9	18

Furthermore, at day 7 after the corneal alkali injury, more WT mice (100%) had achieved complete corneal re-epithelialization than Akita mice (88.9%) (**Figure 3B**; **Table 2**); however, this difference was not statistically significant (*n* = 9, *p* > 0.9999).

Slit lamp images of corneas from the left uninjured eye of the mice showed that no impairment was present at baseline, day 3, and day 7 after injury of the corneas from the right eyes for both WT and Akita mice. Bright-field images showed transparent corneas with no epithelial defect for all corneas (**Supplementary Figure 1A**), whereas fluorescein stain under cobalt blue light indicated that the cornea showed no absorption of the dye, meaning that the corneas were intact (**Supplementary Figure 1B**)

**Figure 4:**
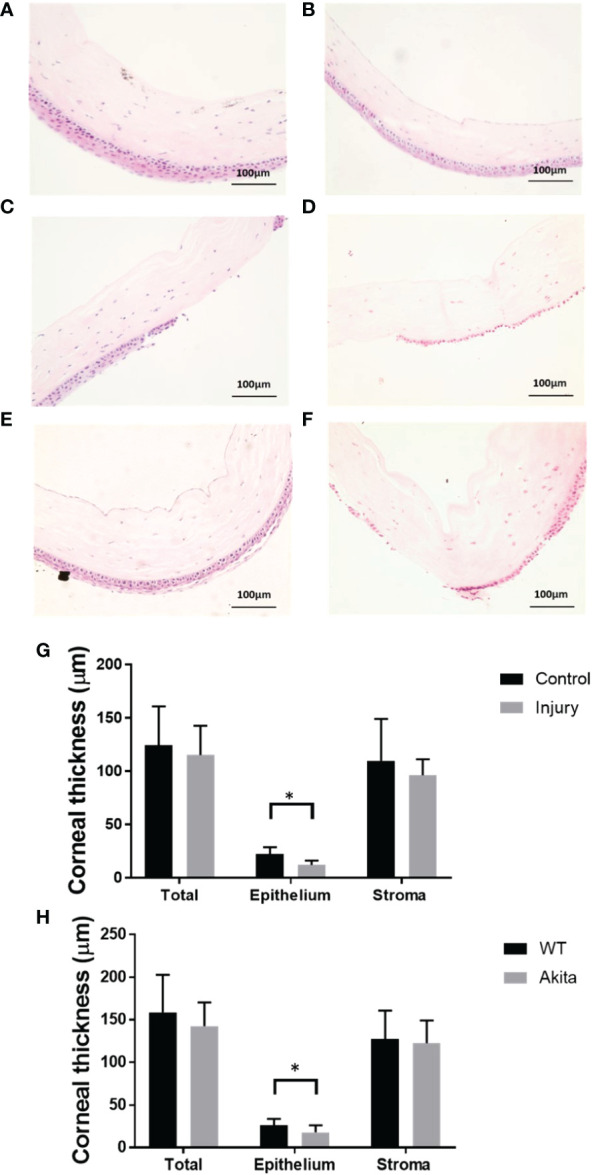
H&E staining of the cornea cross section and thickness measurements. Representative images of corneas were selected from **(A)** a WT mouse at baseline, **(B)** an Akita mouse at baseline, **(C)** a WT mouse immediately after corneal alkaline injury, **(D)** an Akita mouse immediately after corneal alkaline injury, **(E)** a WT mouse immediately at day 3 after corneal alkaline injury, and **(F)** an Akita mouse immediately at day 3 after corneal alkaline injury. **(G)** Corneal thickness measurement for total cornea, corneal epithelium, and corneal stroma for mouse corneas without injury (control group) and those immediately after injury; significantly reduced cornea thicknesses were found in the injured group compared with controls (*n* = 7, *p* = 0.0129). **(H)** Corneal thickness measurement for total cornea, corneal epithelium, and corneal stroma for corneas from WT mice and Akita mice at day 3 after injury. Significantly reduced cornea thickness was found for Akita mice at day 3 after injury compared to the WT group (*n* = 7 for both the WT and Akita groups, *p* = 0.0498). *p<0.05.

To further characterize the effectiveness of the mouse corneal alkaline burn model, hematoxylin and eosin (H&E) staining was conducted on corneal sections, and there was a significantly reduced corneal epithelium thickness between the injury and control group (*n* = 7, *p* = 0.0129), while no significant changes in thickness were found between the injured and the uninjured group in total corneal thickness (*n* = 7, *p* = 0.6725) and stromal thickness (*n* = 7, *p* = 0.4906) (**Figure 4E**). Meanwhile, there was no significant changes between epithelial thickness of WT and Akita mice at baseline (**Figures 4A, B**).

To further confirm the delayed cornea wound healing in Akita mice, cornea thickness was checked between the WT and Akita groups at day 3 after injury. There was significantly reduced cornea epithelium thickness at day 3 after injury (*n* = 7, *p* = 0.0498), whereas no significant changes were found between the WT and Akita groups in total corneal thickness (*n* = 7, *p* = 0.4586) and stromal thickness (*n*= 7, *p* = 0.3654) (**Figures 4C, D, F**).

### 3.3 Tear protein analysis of diabetic and WT mice at baseline and after corneal alkaline burn injury

#### 3.3.1 Diabetic mice had altered baseline tear protein chemokine and growth factor levels compared to wild-type mice

**Figure 5:**
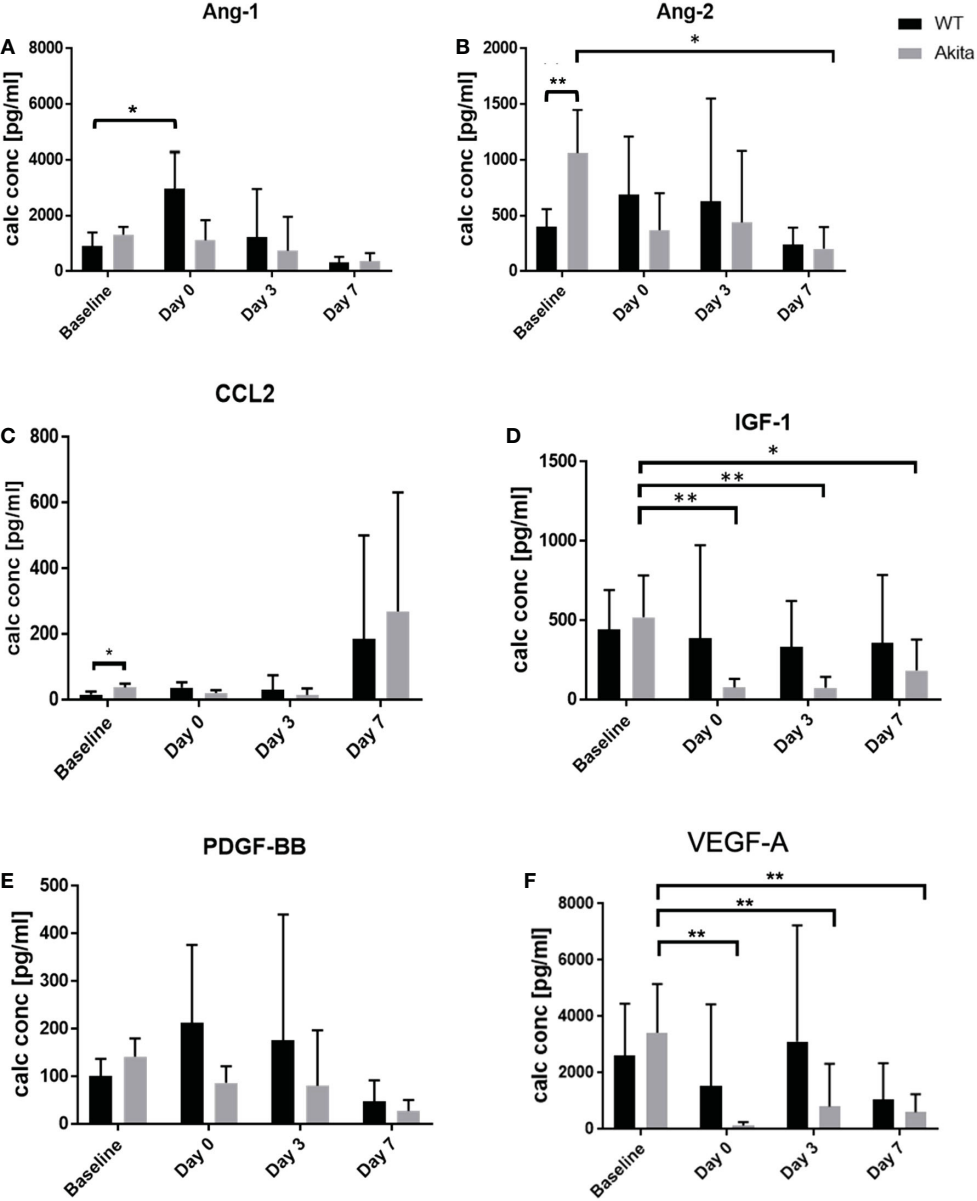
Comparison of tear protein concentration of the right (injured) eye from WT and Akita mice measured at baseline (before injury) with day 0, day 3, and day 7 after corneal alkaline injury. **(A)** Tear level of Ang-1 showed significantly elevated level immediately after injury WT mice (*n* = 6, *p* = 0.0412). **(B)** Tear level of Ang-2 showed significantly higher tear level at baseline and has significantly decreased at day 7 after injury for Akita mice (*n* = 6, *p* = 0.0243). **(C)** Tear level of CCL2 showed significantly elevated level in Akita mice at baseline. **(D)** Tear level of IGF-1 has significantly decreased immediately after injury (*n* = 8, *p* = 0.0018), and on post-injury day 3 (*n* = 8, *p* = 0.0026) and day 7 (*n* = 8, *p* = 0.0147) in Akita mice. **(E)** Tear level of PDGF-BB showed a trend of decrease from baseline level to the post-injury period in tear levels of Akita mice; however, there was no statistical significance. **(F)** Tear level of VEGF-A for Akita mice has significantly decreased immediately after injury (*n* = 8, *p* = 0.0016), on day 3 (*n* = 8, *p* = 0.0063), and on post-injury day 7 (*n* = 8, *p* = 0.0032) compared to baseline level (*n* number means the number of eyes/mice from both the WT and Akita groups, same for all measurements). *p<0.05; **p<0.01.

To analyze the level of cytokine secretion on the ocular surface, tear samples were collected at baseline, day 0, day 3, and day 7 after the corneal alkaline burn, and a protein micro-array assay was conducted with a panel of cytokines related to ocular surface immunity, angiopoietin-1 (Ang-1), angiopoietin-2 (Ang-2), C-C motif chemokine ligand 2 (CCL2), insulin growth factor-1 (IGF-1), platelet-derived growth factor (PDGF), and vascular endothelial growth factor A (VEGF-A) (**Supplementary Table 6**). At baseline before injury, tear concentration of the Ang-2 (1,062 ± 371.5 vs. 402.4 ± 69.35 pg/ml, *p* = 0.0075, *n* = 6) was significantly higher in Akita mice compared to WT mice (**Figure 5B**). In addition, CCL2 showed elevated tear level in Akita mice compared to WT mice (37.9 ± 5.253 vs. 15.9 ± 4.687 pg/ml, *p* = 0.0189, *n* = 6) at baseline (**Figure 5C**). Yet, no significant difference was found for baseline levels of Ang-1, IGF-1, PDGF, and VEGF-A in Akita mice tear samples as compared to WT ones, as indicated by an unpaired *t*-test (**Figures 5A, C–F**).

#### 3.3.2 Diabetic mice had significant impairment of tear growth factor and chemokine secretion after corneal alkaline burn injury

To compare whether there is a significant change of tear protein level at day 0, day 3, and day 7 after injury as compared with baseline level, one-way ANOVA was performed to compare the tear levels of Ang-1, Ang-2, CCL2, IGF-1, PDGF, and VEGF-A at baseline with each post-injury time point. For WT samples, the result showed no significant changes in tear levels of Ang-2, CCL2, IGF-1, PDGF, and VEGF-A between baseline and at day 0, day 3, and day 7, respectively, after the corneal alkaline burn **Supplementary Tables 2–6** **)**. However, tear Ang-1 showed an increased level from 910.5 ± 216.3 pg/ml to 2,967 ± 1,337 pg/ml at day 0 after injury in WT mice (*p* = 0.0412, *n* = 8), but not in Akita mice. Among the result presented for Akita mice samples, there was a significant decrease in tear levels of Ang-2, IGF-1, and VEGF-A immediately (day 0) after the chemical injury in comparison with baseline measurement (**Supplementary Tables 2, 4, 6)** (**Figures 5B, D, F**). In particular, the tear IGF-1 level from Akita mice decreased from a baseline level of 520.1 ± 118.3 pg/ml to 77.65 ± 22.04 pg/ml (*p* = 0.0018, *n* = 8), whereas the tear VEGF-A level from Akita mice decreased from 3,423 ± 772.8 pg/ml to 143.3 ± 59.82 pg/ml (*p* = 0.0016, *n* = 8). Yet, no significant changes were found in tear samples of WT mice immediately after injury except for Ang-1.

At day 7 after injury, the tear concentration of Ang-2 decreased from a baseline level of 1,062 ± 172.4 pg/ml to 201.7 ± 87.21 pg/ml (*p* = 0.0243, *n* = 6) (**Figure 5D**; **Supplementary Table 2**). Meanwhile, there was also a significant decrease in tear levels of IGF-1 and VEGF-A both at day 3 after the injury and at day 7 after the CABI in Akita mice, in comparison with the baseline level (**Figures 5D; F**; **Supplementary Tables 4, 6**). The tear concentration of IGF-1 in Akita mice decreased from a baseline level of 520.1 ± 118.3 pg/ml to 75.46 ± 31.04 pg/ml at day 3 after injury (*p* = 0.0026, *n* = 8) and to 185.7 ± 72.96 at day 7 after the injury (*p* = 0.0147, *n* = 8). Additionally, the tear concentration of VEGF-A in Akita mice decreased from a baseline level of 3,423 ± 772.8 pg/ml to 805.6 ± 573.1 pg/ml at day 3 after injury (*p* = 0.0063, *n* = 8) and to 601.6 ± 243.8 pg/ml at day 7 after injury (*p* = 0.0032, *n* = 8) (**Figure 5F**; **Supplementary Table 6**). Although without significance, the tear CCL2 level in diabetic mice decreased from a baseline level of 37.9 ± 5.253 pg/ml to a day 3 level of 14.1 ± 9.15 pg/ml (*p* = 0.1308, *n* = 8), whereas no apparent decrease was observed for WT mice (*p* = 0.9991, *n* = 8) (**Figure 5C**; **Supplementary Table 3**). Furthermore, in diabetic Akita mice, there was also a trend of decrease of tear expression of Ang-2 at day 0 and day 3 after injury and of PDGF-BB at day 0, day 3, and day 7 after injury (**Figures 5B, E**; **Supplementary Tables 2, 5**).

### 3.4 Diabetic mice showed altered intestinal microbiome composition compared to WT mice

**Figure 6:**
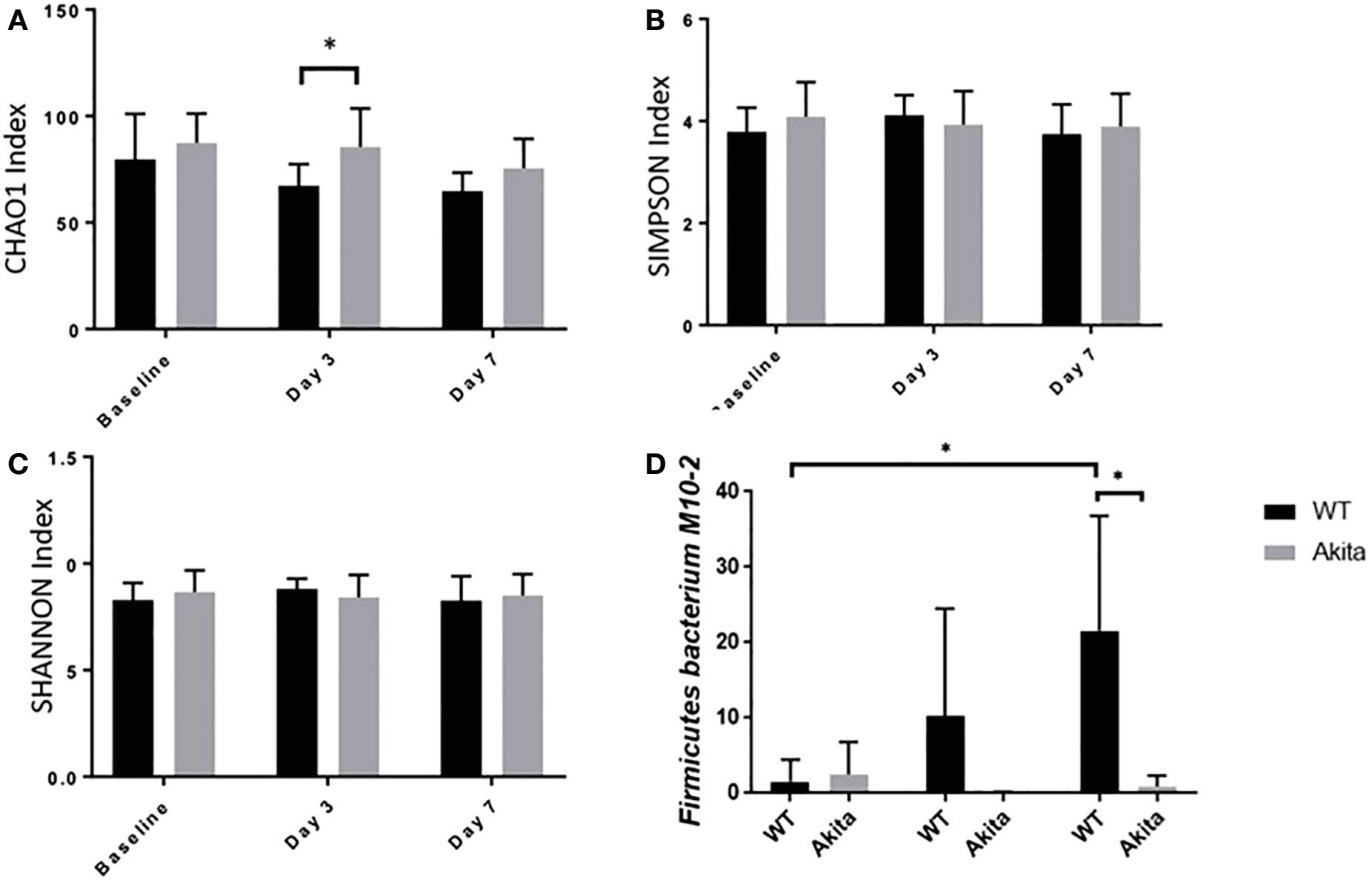
Comparison of intestinal microbiome diversity pattern of WT and Akita mice measured at baseline (before injury) with day 0, day 3, and day 7 aftercorneal alkaline injury. **(A)** Comparison of alpha diversity index at baseline, day 3, and day 7 after injury. Akita mice were found to have higher abundance ofmicrobiota composition as compared to WT mice from the CHAO1 index at day 3 after injury (n = 7, p = 0.0376). **(B, C)** Comparison of alpha diversity index at baseline, day 3, and day 7 after injury by the SIMPSON index **(B)** and SHANNON index **(C)** at day 3 after injury. No significance difference was found at baseline, day 3, or day 7 after injury after one-way ANOVA test (*n* = 7). **(D)** *Firmicutes bacterium M10-2* had higher abundance in WT mice as compared to diabetic mice at day 7 after cornea alkaline injury (*n* = 5, *p* = 0.0164); there was a significant increase of Firmicutes bacterium M10-2 at day 7 after injury as compared to baseline level for WT mice (*n* = 5, *p* = 0.0215) (*n* number means the number of mice from both the WT and Akita groups, same for all measurements). *p<0.05.

The microbiome diversity pattern was compared between groups using an unpaired *t*-test of the CHAO1, Simpson, and Shannon indices at baseline, day 3, and day 7 after the corneal alkali injury. Akita mice were found to have higher diversity in microbiota composition at baseline (*n* = 7, *p* = 0.0370) and day 3 (*n* = 7, *p* = 0.0376) after corneal alkaline injury, as compared to WT mice from the CHAO1 index (**Figure 6A**), whereas the difference was insignificant with the Shannon and Simpson indices (**Figures 6B, C**). At day 7 after the alkaline injury, the difference in gut microbiome diversity was insignificant between WT and Akita mice with the CHAO1, Shannon, and Simpson index (*n* = 7, *p* = 0.1048) (**Figure 6A**).

Additionally, longitudinal changes in gut microbiome diversity pattern at day 3 and day 7 after injury were measured. The results showed that Akita mice have significantly reduced gut microbiota diversity at day 3 in the post-injury period compared to baseline from the CHAO1 index (*n* = 7, *p* = 0.0137) (**Figure 6A**) based on a one-way ANOVA test (*p* = 0.2894). Comparatively, there were no significant changes in gut microbiome diversity pattern in WT mice over time (**Figure 6A**).

**Figure 7:**
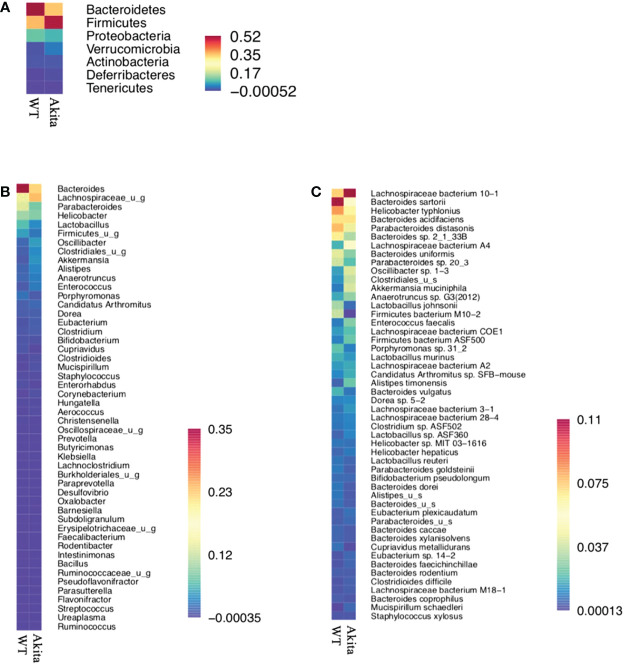
Heat maps for relative abundance of bacteria present in the intestinal microbiome of WT and Akita mice at the phylum, species, and genus level. **(A)** Heat map of bacteria present in intestinal microbiome of WT and Akita mice generated at the phylum level based on mean relative abundance. **(B)** Heat map of bacteria present in the intestinal microbiome of WT and Akita mice generated at the genus level based on mean relative abundance. **(C)** Heat map of bacteria present in the intestinal microbiome of WT and Akita mice generated at the species level based on mean relative abundance. Values of relative abundance are presented with corresponding colors as shown in the legend for B to D (*n* = 5 in WT and Akita).

Individually, *Firmicutes bacterium M10-2* was found to be significantly more abundant in WT mice at day 7 after the corneal alkaline injury (*n* = 5, *p* = 0.0164), while an increase in abundance level was observed at day 7 after injury in WT mice only, but not in Akita mice (*n* = 5, *p* = 0.0215) (**Figure 6D**).The means of relative abundance of bacteria present in the intestinal microbiome of WT and Akita mice were acquired at the phylum, genus, and species level and compared in the form of heat maps, as shown in **Figures 7B, C**. At the phylum level, Bacteroidetes were in higher relative abundance of 0.52 in WT mice as compared to Akita mice, with a relative abundance of 0.35. Conversely, Firmicutes were in lower relative abundance at 0.35 in WT mice as compared to Akita mice with a relative abundance of 0.52. Additionally, Proteobacteria were in higher relative abundance in WT mice compared to Akita mice whereas Verrucomicrobia were in higher relative abundance in Akita mice compared to WT mice. There were no significant differences in relative abundance between WT and Akita mice for Actinobacteria, Deferribacteres, and Tenericutes (*n* = 5) (**Figure 7A**).

At the genus level, Bacteroides were in higher relative abundance at 0.35 in WT mice than in Akita mice at 0.23; Lachnospiraceae_u_g were in higher relative abundance at 0.23 in Akita mice than in WT mice at 0.12; Parabacteroides, Lactobacillus, and Firmicutes_u_g were all in higher abundance in WT mice than in Akita mice (*n* = 5) (**Figure 7B**).

At the species level, *Bacteroides sartorii*, *Helicobacter typhlonius*, *Parabacteroides distasonis*, *Bacteroides* sp. *2_1_33B*, *Parabacteroides* sp. *20_3*, *Bacteroides uniformis*, *Lactobacillus johnsonii*, and *Firmicutes bacterium M10-2* were in higher relative abundance in WT mice than in Akita mice, whereas *Akkermansia muciniphila*, *Lachnospiraceae bacterium 10-1*, *Lachnospiraceae bacterium A4*, *Oscillibacter* sp. *1-3*, and Clostridioides were in higher relative abundance in Akita mice compared to WT mice (*n* = 5) (**Figure 7C**).

### 3.5 Diabetic mice showed altered T-cell profile at baseline and on post-injury day 3

#### 3.5.1 Diabetic mice exhibited an impaired systemic adaptive immune response towards cornea injury compared to wild-type mice

**Figure 8:**
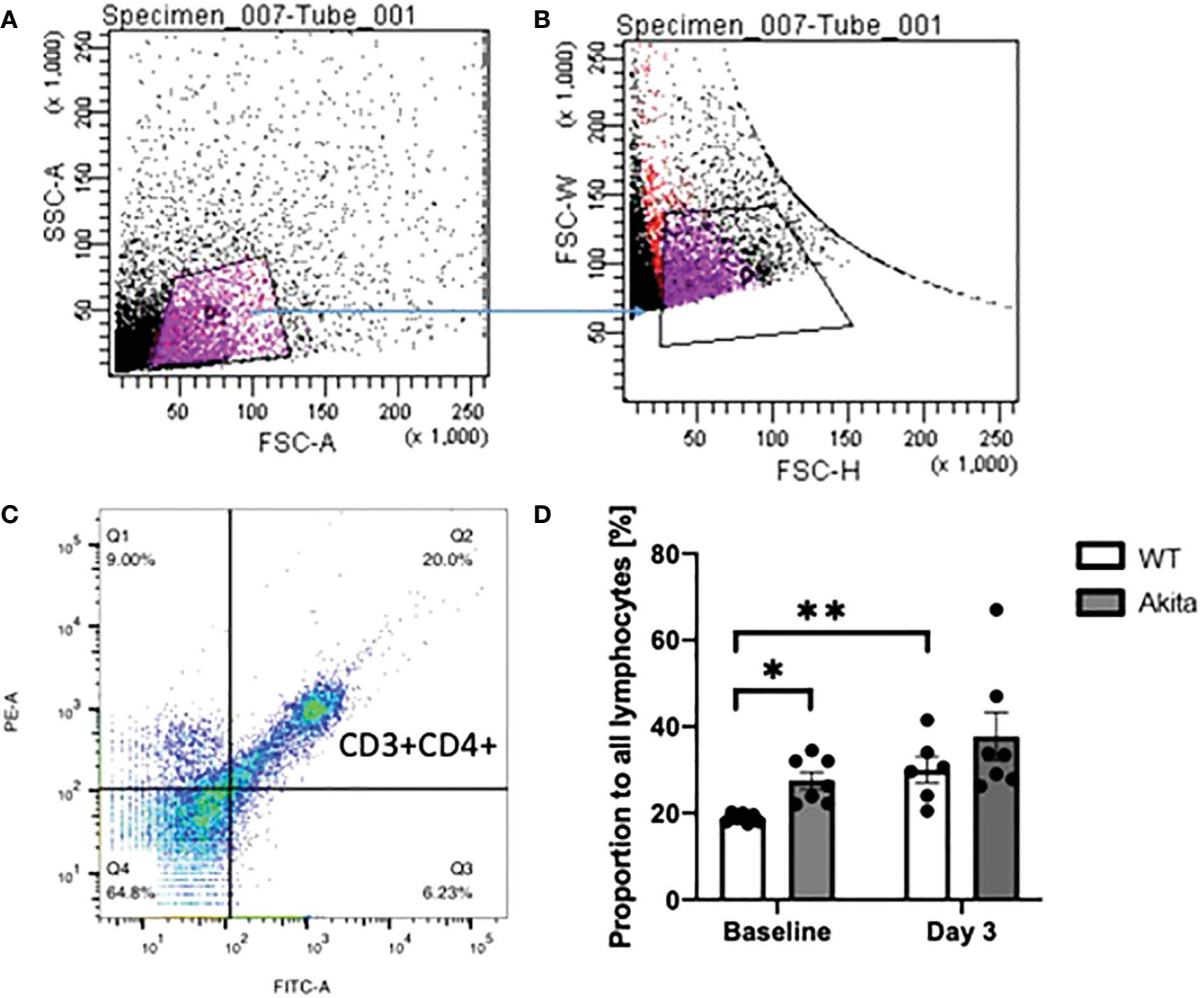
Proportion of CD3+CD4+ T cells in lymphocytes in peripheral blood samples of WT mice and Akita mice at baseline and at day 3 after corneal alkaline injury **(A)** Gating of lymphocytes (P1). **(B)** Gating of single cells against double or multiple cells (P2). **(C)** Gating of CD3+CD4+ T cells. **(D)** Akita mice had a higher abundance of CD3+CD4+ T cells in peripheral blood as compared to WT mice at baseline as indicated by an unpaired *t*-test (*n* = 7, *p* = 0.0146). WT mice had a significant increase of CD3+CD4+ cells in the peripheral blood at day 3 after the injury as indicated by a paired *t*-test (*n* = 7 for both the WT and Akita groups, *p* = 0.0022). *p<0.05; **p<0.01.

Peripheral blood samples of WT and Akita mice were collected at baseline and day 3 after injury and flow cytometry was used to analyze the levels of CD3+CD4+ T cells. All gates were set on the FlowJo software, with P1 gating for lymphocytes, P2 gating for single cells against doublets or clusters of multiple cells, and Q2 gating for both FITC and PE fluorescently labeled cells (**Figures 8A–C**). The proportions of CD3+CD4+ cells from WT and Akita mice at baseline were analyzed by an unpaired *t*-test with a significance level of 0.05. Akita mice were found to have a significantly greater proportion of CD3+CD4+ cells as compared to WT mice at baseline (*n* = 7, *p* = 0.0146) (**Figure 8D**). However, the difference between groups became insignificant at day 3 after injury (**Figure 8D**). Additionally, the proportions of CD3+CD4+ cells from WT and Akita mice at baseline were compared by a paired *t*-test with a significance level of 0.05 for longitudinal analysis. WT mice had significantly increased CD3+CD4+ cells in the peripheral blood at day 3 after injury compared to baseline (*n* = 7, *p* = 0.0022), whereas Akita mice had no significant changes in CD3+CD4+ cells in the peripheral blood before and after injury (**Figure 8D**).

#### 3.5.2 Diabetic mice exhibited an impaired ocular surface adaptive immune response towards cornea injury compared to WT mice

**Figure 9:**
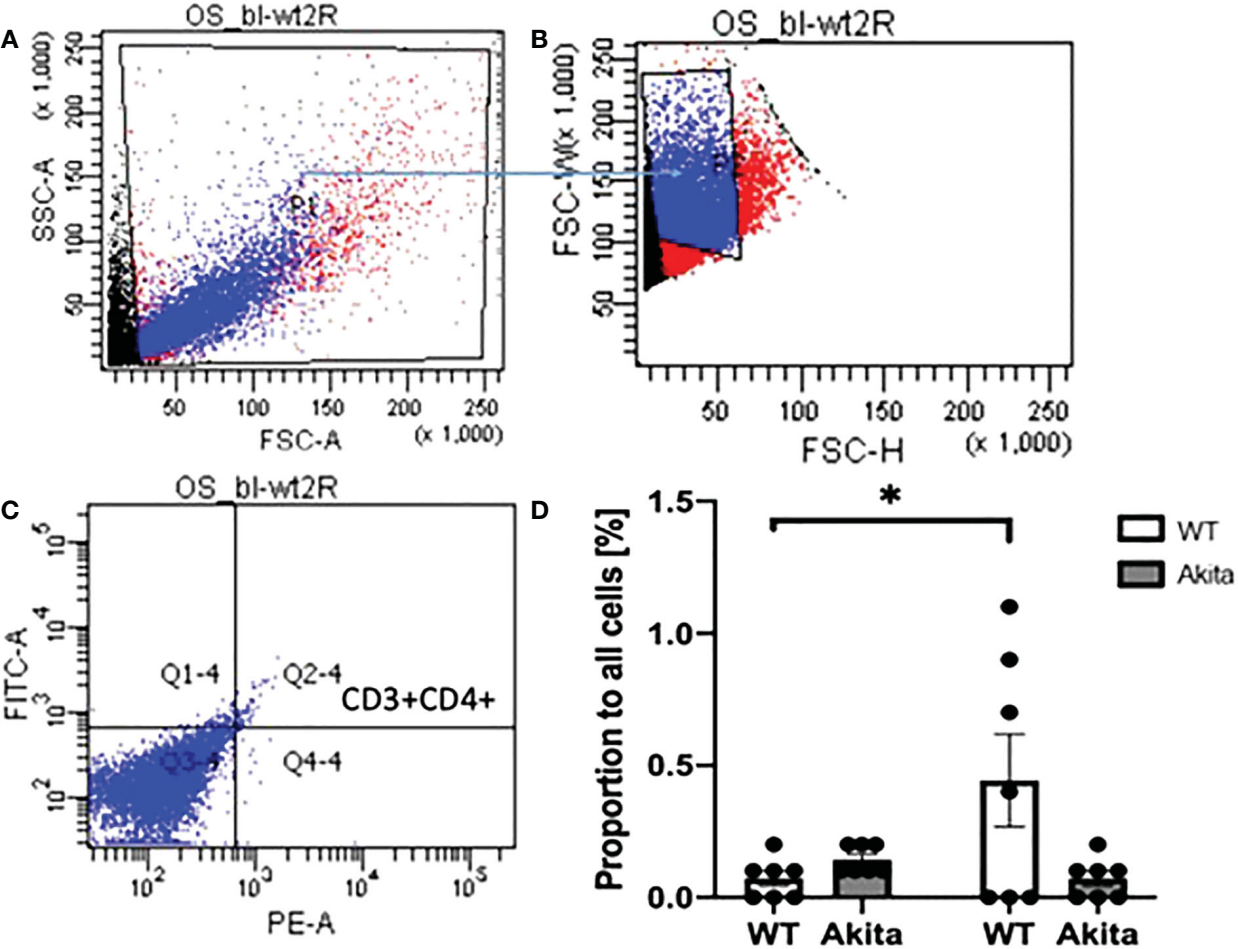
Proportion of CD3+CD4+ T cells in lymphocytes in ocular surface samples of WT and Akita mice at baseline and day 3 after corneal alkaline injury. **(A)** Gating of lymphocytes (P1). **(B)** Gating of single cells against double or multiple cells (P2). **(C)** Gating of CD3+CD4+ T cells (Q2–4). **(D)** Using a paired *t*-test, WT mice had a significant increase of CD3+CD4+ T cells on the ocular surface at day 3 after CABI as compared to baseline (*n* = 7, *p* = 0.0471), whereas no significant change was observed in CD3+CD4+ T cells on Akita mice ocular surface after injury (*n* = 7 for both the WT and Akita groups, *p* = 0.0941). *p<0.05.

In addition, the proportion of CD4+CD3+ T cells were tested with ocular surface samples taken from the mice at day 3 after the chemical injury. The gates were set on FlowJo as shown in **Figures 9A–C**. Using a paired *t*-test, we found that WT mice have a significant increase of CD3+CD4+ T cells on the ocular surface at day 3 after corneal alkali injury as compared to baseline (*n* = 7, *p* = 0.0471), whereas no such significant change was found in CD3+CD4+ T cells on the ocular surface of Akita mice after injury (*n* = 7, *p* = 0.0941) (**Figure 9D**). Moreover, Akita mice showed more abundant CD3+CD4+ T cells as compared to WT mice at baseline, yet the difference is insignificant using an unpaired *t*-test (*n* = 7, *p* = 0.0639) (**Figure 9D**).

## 4 Discussion

### 4.1 We report a highly repeatable animal model of diabetic cornea wound healing

For our experiments, the Akita mouse was selected to model a persistent hyperglycemic state. This is an animal model of T1DM that exhibits significant hyperglycemia starting from 3 to 4 weeks of age. While it has been previously used as an animal model of diabetic retinopathy complications, our group is the first to use this model to investigate impaired cornea wound healing after injury in diabetic mice versus controls (24). In our experiments, Akita mice showed significantly slower corneal re-epithelialization rates compared to WT mice after alkaline chemical injury. This is consistent with findings from diabetic cornea wound healing studies using other types of animal models (25). Meanwhile, the result also demonstrates that alkaline burn injury induced on Akita mouse cornea is an excellent model to investigate molecular pathogenic and therapeutic aspects of corneal wound healing in diabetes.

### 4.2 Diabetic mice exhibited impaired ocular surface immune response to corneal injury

The results of protein micro-array assay of tear cytokines reported that diabetic mice had reduced tear levels of VEGF-A, Ang2, and IGF-1 after injury compared to baseline. The components of tear fluid are secreted from several sources including conjunctival goblet cells, the lacrimal gland, the cornea epithelium, and the episcleral vascular system in close proximity (26). The altered tear cytokine levels suggest an inability of the ocular surface to mount an immune response to promote cornea wound healing after injury. The importance of VEGF in diabetic complications has been previously comprehensively established (27, 28). VEGF is known to promote vascular endothelial cell proliferation, migration, and vasopermeability. VEGF levels were reported to be elevated in the blood of diabetic patients, playing a key role in diabetes-related morbidity such as diabetic retinopathy, age-related macular degeneration, and a variety of cardiovascular diseases. In ocular surface diseases such as DED, VEGF is known as an inflammatory marker that impairs the surface epithelium of the eye (29). However, VEGF is also recognized as a pleiotropic factor with a broad effect on endothelial, neuronal, and glial behaviors (30). Because of the above-mentioned importance of VEGF in diabetes and in corneal wound healing response, VEGF level was measured in our experiments and was found to be suppressed in diabetic mice after injury, suggesting that while sustained high levels of VEGF in the serum may correlate with the development of microvascular complications, post-injury tear VEGF levels may be important in the cornea wound healing response.

Our study reported an elevated baseline level of CCL2 in tear samples of Akita mice. CCL2, also known as MCP-1, is a proinflammatory chemokine and has a controversial role in diabetes and wound healing. Previous studies suggested that CCL2 is beneficial in wound healing by restoring the macrophage response (31). On the other hand, it is also a risk factor for worsening diabetic nephropathy due to its role in promoting inflammation (32). In previous ocular studies, overexpression of the CCL2 was involved in the pathogenesis of diabetic retinopathy (33). In our experiments, tear secretion of CCL2 diminished sequentially after injury in diabetic mice but not in WT mice, suggesting its importance in the cornea wound healing response.

Previous reports have shown higher blood levels of Ang-2 in diabetic mice compared to controls (34). The function of Ang-2 in the integrity of the blood–retinal barrier was previously highlighted in studies of diabetic retinopathy (35). In our experiments, tear Ang-2 was lower in diabetic mice compared to controls immediately after injury (day 0), suggesting an important role in the initiation of the cornea wound healing response.

The association between IGF and metabolic syndromes has been explored extensively in previous experiments (36). Patients with very high or very low levels of serum IGF-1 are both at increased risk of diabetes (37, 38). Meanwhile, animal studies indicate that IGF-1 has an essential inhibitory effect on cell apoptosis and has been shown to promote wound healing (39, 40). This is consistent with our finding of diminished tear IGF-1 after injury in diabetic mice, confirming its importance in the cornea wound healing response.

Taken together, our findings show that cytokines are key mediators of corneal wound healing after injury, despite the fact that they are normally detrimental in the pathogenesis of common microvascular complications of diabetes, including retinopathy and nephropathy. The fact that tear levels of these cytokines are suppressed in diabetic mice following corneal alkaline burn injury demonstrates the importance of the local inflammatory response in initiating and propagating cornea re-epithelialization.

### 4.3 Altered gut microbial diversity in response to corneal injury and in diabetic mice

In our study, diabetic mice and WT mice each had distinct microbial abundance patterns at baseline. In response to cornea injury, both diabetic and WT mice demonstrated changes in alpha diversity patterns compared to baseline. Such a response has been previously documented in other forms of severe non-abdominal injuries, including traumatic brain injury and spinal cord injury resulting in intestinal dysbiosis, which subsequently was followed by impaired systemic immunity (41, 42). Such changes may represent a stress response resulting in a temporary alteration in gut microbiome composition. This is consistent with previous findings that psychological stress or depression may cause intestinal dysbiosis (43). The resulting altered intestinal microbiome may, in turn, impact the adaptive immune system, making it less effective in promoting healing after an insult.

Previous studies have reported the close association between intestinal dysbiosis and type 2 diabetes. The gut microbiota contributes to maintenance of intestinal integrity by modulating tight junction proteins between gut epithelial cells (44). Alterations in intestinal microbiome have been shown to enhance systemic inflammation (45) due to the increased abundance of pathogenic bacteria. In diabetes, intestinal dysbiosis may further promote microvascular complications through endotoxemia. This can be ameliorated through the use of probiotic treatment, which has been shown to effectively reverse both intestinal dysbiosis and diabetic progression in T2DM (45).

When considering the microbiome response on an individual species level, our study identified several potential targets for manipulation in future studies (46). In our study, the abundance of *Bacteroides* was initially lower in diabetic mice compared to controls at baseline, but was significantly elevated after cornea injury. A previous study demonstrated a reduced abundance of *Bacteroides* in Bio-Breeding diabetes-prone (BB-DP) rats (47). Furthermore, in our experiments, *A. muciniphila* is present only in diabetic mice after the injury and not in controls. In previous studies, *A. muciniphila* was shown to have beneficial effects in attenuating diabetic complications in humans (48). It has also been shown to have a protective role against immune-mediated liver injury in mice (49). We also observed lower abundance levels of *H. typhlonius* in diabetic mice compared to controls. This was consistent with the findings of previous published results in humans (50). Its role in diabetic complications, however, has not been fully elucidated. Finally, the abundance level of *Firmicutes bacterium M10-2* was significantly reduced in diabetic mice after injury. It was previously reported that that the ratio of Firmicutes and Bacteroidetes correlated with body mass index (51). However, no published study has investigated the role of firmicutes bacterium in diabetes pathogenesis and complications.

### 4.4 Diabetic mice have impaired systemic and local immune response to corneal injury

Upon stimulation by the innate immune response, CD4+ cells differentiate into T helper (Th) cells and regulatory T (Treg) cells, facilitating adaptive immunity. Th and Treg cells play important roles in response to insult, including acute kidney injury (52) and skin injury (53).

In our study, Akita mice had higher peripheral blood CD4+ T-cell counts compared to WT mice at baseline. This finding may represent a persistent inflammatory response towards intestinal dysbiosis and endotoxemia in the Akita mice. This, however, changed significantly in Akita and WT mice groups after cornea injury. While WT mice had an upregulation of CD3+CD4+ T-cell count after injury, diabetic mice had no significant changes, both on the ocular surface and in peripheral blood. This highly suggests that diabetic mice are incapable of initiating a proper adaptive immune response towards injury. This finding may partially explain impaired ocular surface immune response and wound healing after injury under diabetic conditions. Furthermore, given our understanding of the link between intestinal microbiome and T-cell-mediated immunity, the differences in immune response between WT and Akita mice may be a consequence of the observed differences in microbiome diversity and abundance patterns.

### 4.5 Limitations of the study

Our study had several limitations. Firstly, in our experiments, we used the Akita mouse as the animal model of diabetes. It is important to note that there are etiological differences between the diabetes in this model and that in human patients, which may serve as confounding factors, particularly in regard to the non-glycemic pathways for pathogenesis and complications of diabetes. It may be worthwhile to confirm whether our observations are present in other animal models of diabetes and cornea injury. Furthermore, differences in the gut microbiome profile between mouse and human serve another limitation, as our target bacteria and pathways may not be applicable to the human gut. Thirdly, this is an observational study rather than a therapeutic trial. We will next need to examine the impact of treating the intestinal dysbiosis in diabetic mice, either through probiotics or fecal transplantation, on cornea wound healing outcomes.

### 4.6 Clinical implications

Our results demonstrated a potential link between gut microbiome, systemic and local immunity, and cornea wound healing, suggesting that further exploration of this relationship in diabetes is worthwhile. Keratopathy is a common and sight-threatening complication of diabetes, manifested by delayed cornea wound healing, recurrent corneal erosion syndrome, and neurotrophic ulcers. Current treatment options are limited and do not address the underlying pathological changes caused by DM. Our continued research in this area may eventually pave the way for specific dietary modifications, fecal transplantation, and/or probiotic therapy as promising and more cost-effective options in ameliorating complications of DM.

## 5 Conclusion

In summary, we demonstrated that in diabetic mice, impaired cornea wound healing was associated with an inability to mount a systemic and local immune response to ocular chemical injury. The lack of ability of the immune system in diabetic mice to respond to the ocular insult, both locally and systemically, was evidenced by a lack of peripheral blood T-cell proliferation and lack of tear chemokine and growth factor production. This is in stark contrast to the immune system in WT controls, where a swift immune response facilitated prompt cornea wound healing. Our study further postulated that baseline differences in intestinal microbial diversity and abundance patterns may account for the observed differences in immune response between diabetic mice and controls. While further in vivo and in vitro experiments are needed to clarify the relationship between gut microbiome and systemic and ocular surface immunity, our results add to the growing amount of evidence of the intimate link between the two systems. The gut microbiome may provide a useful and non-invasive target for manipulation in diabetic patients with reduced morbidity and mortality.

## Data availability statement

The raw data supporting the conclusions of this article will be made available by the authors, without undue reservation.

## Ethics statement

The animal study was reviewed and approved by the Committee on the Use of Live Animals in Teaching and Research (CULATR, 4696-18).

## Author contributions

All authors attest that they meet the current ICMJE criteria for authorship. YB, KS, and AC-YL were involved in the study design, data collection, data analysis, manuscript writing, and editing. JY-KC, HLW, A-KN, TC-YC, VJ, and LT were involved in data collection, data analysis, manuscript writing, and editing. All authors contributed to the article and approved the submitted version.

## References

[1] Schultz RO Van Horn DL Peters MA Klewin KM Schutten WH. Diabetic keratopathy. Trans Am Ophthalmol Soc (1981) 79:180–99. PMC1312182 7342400

[2] Vieira-Potter VJ Karamichos D Lee DJ. Ocular complications of diabetes and therapeutic approaches. BioMed Res Int (2016) 2016:3801570. doi: 10.1155/2016/3801570 27119078 PMC4826913

[3] Shih KC Lam KS Tong L. A systematic review on the impact of diabetes mellitus on the ocular surface. Nutr Diabetes (2017) 7(3):e251. doi: 10.1038/nutd.2017.4 28319106 PMC5380897

[4] Ljubimov AV. Diabetic complications in the cornea. Vision Res (2017) 139:138–52. doi: 10.1016/j.visres.2017.03.002 PMC5660664 28404521

[5] Yang L Di G Qi X Qu M Wang Y Duan H. Substance p promotes diabetic corneal epithelial wound healing through molecular mechanisms mediated *via* the neurokinin-1 receptor. Diabetes (2014) 63(12):4262–74. doi: 10.2337/db14-0163 25008176

[6] Atiba A Wasfy T Abdo W Ghoneim A Kamal T Shukry M. Aloe vera gel facilitates re-epithelialization of corneal alkali burn in normal and diabetic rats. Clin Ophthalmol (Auckland NZ) (2015) 9:2019–26. doi: 10.2147/OPTH.S90778 PMC4630191 26604672

[7] Segre JA. MICROBIOME. microbial growth dynamics and human disease. Sci (New York NY) (2015) 349(6252):1058–9. doi: 10.1126/science.aad0781 26339017

[8] Chu H Khosravi A Kusumawardhani IP Kwon AH Vasconcelos AC Cunha LD. Gene-microbiota interactions contribute to the pathogenesis of inflammatory bowel disease. Sci (New York NY) (2016) 352(6289):1116–20. doi: 10.1126/science.aad9948 PMC4996125 27230380

[9] Sefik E Geva-Zatorsky N Oh S Konnikova L Zemmour D McGuire AM. MUCOSAL IMMUNOLOGY. individual intestinal symbionts induce a distinct population of RORgamma(+) regulatory T cells. Sci (New York NY) (2015) 349(6251):993–7. doi: 10.1126/science.aaa9420 PMC4700932 26272906

[10] Kim J Choi SH Kim YJ Jeong HJ Ryu JS Lee HJ. Clinical effect of IRT-5 probiotics on immune modulation of autoimmunity or alloimmunity in the eye. Nutrients (2017) 9(11):1166. doi: 10.3390/nu9111166 29068389 PMC5707638

[11] Lu LJ Liu J. Human microbiota and ophthalmic disease. Yale J Biol Med (2016) 89(3):325–30. PMC5045141 27698616

[12] Ivanov II Atarashi K Manel N Brodie EL Shima T Karaoz U. et al: Induction of intestinal Th17 cells by segmented filamentous bacteria. Cell (2009) 139(3):485–98. doi: 10.1016/j.cell.2009.09.033 PMC2796826 19836068

[13] Atarashi K Nishimura J Shima T Umesaki Y Yamamoto M Onoue M. et al: ATP drives lamina propria T(H)17 cell differentiation. Nature (2008) 455(7214):808–12. doi: 10.1038/nature07240 18716618

[14] Murphy EA Velazquez KT Herbert KM. Influence of high-fat diet on gut microbiota: a driving force for chronic disease risk. Curr Opin Clin Nutr Metab Care (2015) 18(5):515–20. doi: 10.1097/MCO.0000000000000209 PMC4578152 26154278

[15] Li Q Gao Z Wang H Wu H Liu Y Yang Y. Intestinal immunomodulatory cells (T lymphocytes): A bridge between gut microbiota and diabetes. Mediators Inflammation (2018) 2018:9830939. doi: 10.1155/2018/9830939 PMC5866888 29713241

[16] Shin YJ Hyon JY Choi WS Yi K Chung ES Chung TY. Chemical injury-induced corneal opacity and neovascularization reduced by rapamycin *via* TGF-beta1/ERK pathways regulation. Invest Ophthalmol Vis Sci (2013) 54(7):4452–8. doi: 10.1167/iovs.13-11684 23716625

[17] Nakano Y Uchiyama M Arima T Nagasaka S Igarashi T Shimizu A. PPARalpha agonist suppresses inflammation after corneal alkali burn by suppressing proinflammatory cytokines, MCP-1, and nuclear translocation of NF-kappaB. Molecules (Basel Switzerland) (2018) 24(1). doi: 10.3390/molecules24010114 PMC6337747 30597991

[18] Chen Y Yang W Zhang X Yang S Peng G Wu T. MK2 inhibitor reduces alkali burn-induced inflammation in rat cornea. Sci Rep (2016) 6:28145. doi: 10.1038/srep28145 27329698 PMC4916419

[19] Wang W Tam KC Ng TC Goit RK Chan KLS Lo ACY. Long-term lutein administration attenuates retinal inflammation and functional deficits in early diabetic retinopathy using the Ins2(Akita/+) mice. BMJ Open Diabetes Res Care (2020) 8(1). doi: 10.1136/bmjdrc-2020-001519 PMC7365433 32665315

[20] Wong HL Bu Y Chan YK Shih KC. Lycium barbarum polysaccharide promotes corneal re-epithelialization after alkaline injury. Exp Eye Res (2022) 221:109151. doi: 10.1016/j.exer.2022.109151 35714698

[21] Soria J Acera A Merayo LJ Durán JA González N Rodriguez S. Tear proteome analysis in ocular surface diseases using label-free LC-MS/MS and multiplexed-microarray biomarker validation. Sci Rep (2017) 7(1):17478. doi: 10.1038/s41598-017-17536-2 29234088 PMC5727318

[22] Singh A Schabath R Ratei R Stroux A Klemke CD Nebe T. Peripheral blood sCD3⁻ CD4+ T cells: A useful diagnostic tool in angioimmunoblastic T cell lymphoma. Hematol Oncol (2014) 32(1):16–21. doi: 10.1002/hon.2080 23798351

[23] Yan J Liao C Taylor BP Fontana E Amoretti LA Wright RJ. A compilation of fecal microbiome shotgun metagenomics from hematopoietic cell transplantation patients. Sci Data (2022) 9(1):219. doi: 10.1038/s41597-022-01302-9 35585088 PMC9117330

[24] Barber AJ Antonetti DA Kern TS Reiter CE Soans RS Krady JK. The Ins2Akita mouse as a model of early retinal complications in diabetes. Invest Ophthalmol Visual Sci (2005) 46(6):2210–8. doi: 10.1167/iovs.04-1340 15914643

[25] Xu K Yu FS. Impaired epithelial wound healing and EGFR signaling pathways in the corneas of diabetic rats. Invest Ophthalmol Vis Sci (2011) 52(6):3301–8. doi: 10.1167/iovs.10-5670 PMC3109029 21330660

[26] Chng C-L Seah LL Yang M Shen SY Koh SK Gao Y. Tear proteins calcium binding protein A4 (S100A4) and prolactin induced protein (PIP) are potential biomarkers for thyroid eye disease. Sci Rep (2018) 8(1):16936. doi: 10.1038/s41598-018-35096-x 30446693 PMC6240106

[27] Wirostko B Wong TY Simo R. Vascular endothelial growth factor and diabetic complications. Prog retinal eye Res (2008) 27(6):608–21. doi: 10.1016/j.preteyeres.2008.09.002 18929676

[28] Aiello LP Wong JS. Role of vascular endothelial growth factor in diabetic vascular complications. Kidney Int Supplement (2000) 77:S113–119. doi: 10.1046/j.1523-1755.2000.07718.x 10997700

[29] Laude A Lim JW Srinagesh V Tong L. The effect of intravitreal injections on dry eye, and proposed management strategies. Clin Ophthalmol (Auckland NZ) (2017) 11:1491–7. doi: 10.2147/OPTH.S136500 PMC5566503 28860698

[30] Penn JS Madan A Caldwell RB Bartoli M Caldwell RW Hartnett ME. Vascular endothelial growth factor in eye disease. Prog retinal eye Res (2008) 27(4):331–71. doi: 10.1016/j.preteyeres.2008.05.001 PMC3682685 18653375

[31] Wood S Jayaraman V Huelsmann EJ Bonish B Burgad D Sivaramakrishnan G. Pro-inflammatory chemokine CCL2 (MCP-1) promotes healing in diabetic wounds by restoring the macrophage response. PLos One (2014) 9(3):e91574. doi: 10.1371/journal.pone.0091574 24618995 PMC3950222

[32] Tesch GH. MCP-1/CCL2: a new diagnostic marker and therapeutic target for progressive renal injury in diabetic nephropathy. Am J Physiology-Renal Physiol (2008) 294(4):F697–701. doi: 10.1152/ajprenal.00016.2008 18272603

[33] Taghavi Y Hassanshahi G Kounis NG Koniari I Khorramdelazad H. Monocyte chemoattractant protein-1 (MCP-1/CCL2) in diabetic retinopathy: latest evidence and clinical considerations. J Cell Commun Signal (2019) 13:451–462. doi: 10.1007/s12079-018-00500-8 PMC6946768 30607767

[34] Lim HS Lip GY Blann AD. Angiopoietin-1 and angiopoietin-2 in diabetes mellitus: relationship to VEGF, glycaemic control, endothelial damage/dysfunction and atherosclerosis. Atherosclerosis (2005) 180(1):113–8. doi: 10.1016/j.atherosclerosis.2004.11.004 15823283

[35] Rangasamy S Srinivasan R Maestas J McGuire PG Das A. A potential role for angiopoietin 2 in the regulation of the blood-retinal barrier in diabetic retinopathy. Invest Ophthalmol Visual Sci (2011) 52(6):3784–91. doi: 10.1167/iovs.10-6386 PMC3109054 21310918

[36] Friedrich N Thuesen B Jorgensen T Juul A Spielhagen C Wallaschofksi H. The association between IGF-I and insulin resistance: A general population study in Danish adults. Diabetes Care (2012) 35(4):768–73. doi: 10.2337/dc11-1833 PMC3308317 22374641

[37] van der Klaauw AA Biermasz NR Feskens EJ Bos MB Smit JW Roelfsema F. The prevalence of the metabolic syndrome is increased in patients with GH deficiency, irrespective of long-term substitution with recombinant human GH. Eur J Endocrinol (2007) 156(4):455–62. doi: 10.1530/EJE-06-0699 17389460

[38] Melmed S. Medical progress: Acromegaly. New Engl J Med (2006) 355(24):2558–73. doi: 10.1056/NEJMra062453 17167139

[39] Emmerson E Campbell L Davies FC Ross NL Ashcroft GS Krust A. Insulin-like growth factor-1 promotes wound healing in estrogen-deprived mice: New insights into cutaneous IGF-1R/ERalpha cross talk. J Invest Dermatol (2012) 132(12):2838–48. doi: 10.1038/jid.2012.228 22810305

[40] Achar RA Silva TC Achar E Martines RB Machado JL. Use of insulin-like growth factor in the healing of open wounds in diabetic and non-diabetic rats. Acta cirurgica Bras (2014) 29(2):125–31. doi: 10.1590/S0102-86502014000200009 24604317

[41] Celorrio M Abellanas MA Rhodes J Goodwin V Moritz J Vadivelu S. Gut microbial dysbiosis after traumatic brain injury modulates the immune response and impairs neurogenesis. Acta Neuropathologica Commun (2021) 9(1):40. doi: 10.1186/s40478-021-01137-2 PMC7944629 33691793

[42] Valido E Bertolo A Fränkl GP Itodo OA Pinheiro T Pannek J. Systematic review of the changes in the microbiome following spinal cord injury: animal and human evidence. Spinal Cord (2022) 60(4):288–300. doi: 10.1038/s41393-021-00737-y 34992210 PMC8989678

[43] Madison A Kiecolt-Glaser JK. Stress, depression, diet, and the gut microbiota: human-bacteria interactions at the core of psychoneuroimmunology and nutrition. Curr Opin Behav Sci (2019) 28:105–10. doi: 10.1016/j.cobeha.2019.01.011 PMC7213601 32395568

[44] Sharma S Tripathi P. Gut microbiome and type 2 diabetes: where we are and where to go? J Nutr Biochem (2019) 63:101–8. doi: 10.1016/j.jnutbio.2018.10.003 30366260

[45] Sanz Y Rastmanesh R Agostoni C. Understanding the role of gut microbes and probiotics in obesity: How far are we? Pharmacol Res (2013) 69(1):144–55. doi: 10.1016/j.phrs.2012.10.021 23147032

[46] Han H Li Y Fang J Liu G Yin J Li T. Gut microbiota and type 1 diabetes. Int J Mol Sci (2018) 19(4). doi: 10.3390/ijms19040995 PMC5979537 29584630

[47] Roesch LF Lorca GL Casella G Giongo A Naranjo A Pionzio AM. Culture-independent identification of gut bacteria correlated with the onset of diabetes in a rat model. Isme J (2009) 3(5):536–48. doi: 10.1038/ismej.2009.5 PMC2972309 19225551

[48] Mithieux G. Does akkermansia muciniphila play a role in type 1 diabetes? Gut (2018) 67(8):1373–4. doi: 10.1136/gutjnl-2017-315732 29440234

[49] Wu W Lv L Shi D Ye J Fang D Guo F. Protective effect of akkermansia muciniphila against immune-mediated liver injury in a mouse model. Front Microbiol (2017) 8:1804. doi: 10.3389/fmicb.2017.01804 29033903 PMC5626943

[50] Dingemanse C Belzer C van Hijum SA Gunthel M Salvatori D den Dunnen JT. Akkermansia muciniphila and helicobacter typhlonius modulate intestinal tumor development in mice. Carcinogenesis (2015) 36(11):1388–96. doi: 10.1093/carcin/bgv120 26320104

[51] Larsen N Vogensen FK van den Berg FW Nielsen DS Andreasen AS Pedersen BK. Gut microbiota in human adults with type 2 diabetes differs from non-diabetic adults. PLos One (2010) 5(2):e9085. doi: 10.1371/journal.pone.0009085 20140211 PMC2816710

[52] Dellepiane S Leventhal JS Cravedi P. T Cells and acute kidney injury: A two-way relationship. Front Immunol (2020) 11. doi: 10.3389/fimmu.2020.01546 PMC7379378 32765535

[53] Boothby IC Cohen JN Rosenblum MD. Regulatory T cells in skin injury: At the crossroads of tolerance and tissue repair. Sci Immunol (2020) 5(47). doi: 10.1126/sciimmunol.aaz9631 PMC7274208 32358172

