# Supplementary material for: The association between altered intestinal microbiome, impaired systemic and ocular surface immunity, and impaired wound healing response after corneal alkaline-chemical injury in diabetic mice
Source: Front Immunol. 2023 Jan 31;14:1063069. doi: 10.3389/fimmu.2023.1063069 (PMC9927643; doi:10.3389/fimmu.2023.1063069)
Supplement: Supplementary file 1 [file Table_1.docx]

**Supplementary Tables**

Supplementary Table 1. Tear protein concentration of Ang-1 of the right (injured) eye measured at baseline (before injury), day 0, day 3 and day 7 after the injury for WT and Akita mice, respectively, n=6

| **Ang-1 concentration [pg/ml]**  **Mean+SEM^1^** | **Baseline** | **Day 0** | **p1** | **Day 3** | **p2** | **Day 7** | **p3** |
| --- | --- | --- | --- | --- | --- | --- | --- |
| **WT** | 910.5 ± 216.3 | 2967 ± 1337 | **0.0412** | 738.5 ± 545.9 | 0.9964 | 365.6 ± 127.4 | 0.9796 |
| **Akita** | 1310 ± 167 | 1110 ± 324.4 | 0.9846 | 1215 ± 867.2 | 0.7513 | 308.6 ± 120.5 | 0.3822 |
| **p4** | 0.6297 | 0.2489 | / | 0.6421 | / | 0.7757 | / |

1. SEM: standard error of the mean

Supplementary Table 2. Tear protein concentration of Ang-2 of the right (injured) eye measured at baseline (before injury), day 0, day 3 and day 7 after the injury for WT and Akita mice, respectively, n=6, At baseline, there is significantly higher tear level of Ang-2 in Akita mice than in WT mice

| **Ang-2 concentration [pg/ml]**  **Mean+SEM** | **Baseline** | **Day 0** | **p1** | **Day 3** | **p2** | **Day 7** | **p3** |
| --- | --- | --- | --- | --- | --- | --- | --- |
| **WT** | 402.4 ± 69.35 | 687.9 ± 233.1 | 0.8220 | 699.6 ± 270 | 0.9139 | \| 204.1  ± 72.93 \| \| --- \| \|  \| | 0.9651 |
| **Akita** | 1062 ± 172.4 | 369 ± 148.2 | 0.0815 | 574.9 ± 447.5 | 0.1308 | 529 ± 156.2 | **0.0243** |
| **p4** | **0.0075** | 0.2816 | / | 0.7250 | / | 0.7640 | / |

Supplementary Table 3. Tear protein concentration of CCL2 of the right (injured) eye measured at baseline (before injury), day 0, day 3 and day 7 after the injury for WT and Akita mice, respectively, n=8, At baseline, there is significantly higher tear level of CCL2 in Akita mice than in WT mice

| **CCL2**  **concentration [pg/ml]**  **Mean+SEM** | **Baseline** | **Day 0** | **p1** | **Day 3** | **p2** | **Day 7** | **p3** |
| --- | --- | --- | --- | --- | --- | --- | --- |
| **WT** | 15.9 ± 4.687 | 36.83 ± 9.864 | 0.9977 | 31.71 ± 25.48 | 0.9991 | 186.1±181.6 | 0.4837 |
| **Akita** | 37.9 ± 5.253 | 20.1 ± 6.5 | 0.9996 | 14.1 ± 9.15 | 0.1308 | 268.8 ± 162.4 | 0.3129 |
| **p4** | **0.0189** | 0.3070 | / | 0.4721 | / | 0.7558 | / |

Supplementary Table 4. Tear protein concentration of IGF-1 of the right (injured) eye measured at baseline (before injury), day 0, day 3 and day 7 after the injury for WT and Akita mice, respectively, n=8

| **IGF-1 concentration [pg/ml]**  **Mean+SEM** | **Baseline** | **Day 0** | **p1** | **Day 3** | **p2** | **Day 7** | **p3** |
| --- | --- | --- | --- | --- | --- | --- | --- |
| **WT** | 495.4 ± 127 | 410.3 ± 337.2 | 0.9928 | 401.7 ± 140.9 | 0.9905 | 437.3 ± 225.8 | 0.9977 |
| **Akita** | 520.1 ± 118.3 | 77.65 ± 22.04 | **0.0018** | 75.46 ± 31.04 | **0.0026** | 185.7 ± 72.96 | **0.0147** |
| **p4** | 0.6527 | 0.2246 | / | 0.0952 | / | 0.3621 | / |

Supplementary Table 5. Tear protein concentration of PDGF-BB of the right (injured) eye measured at baseline (before injury), day 0, day 3 and day 7 after the injury for WT and Akita mice, respectively, n=8

| **PDGF-BB**  **concentration [pg/ml]**  **Mean+SEM** | **Baseline** | **Day 0** | **p1** | **Day 3** | **p2** | **Day 7** | **p3** |
| --- | --- | --- | --- | --- | --- | --- | --- |
| **WT** | 101.6 ± 17.72 | 212.6 ± 82.03 | 0.7319 | 175.6 ± 152.7 | 0.9162 | 47.17 ± 25.81 | 0.9637 |
| **Akita** | 141.6 ± 21.93 | 86.9 ± 19.9 | 0.8045 | 80.82 ± 51.96 | 0.6856 | 27.6 ± 11.52 | 0.2429 |
| **p4** | 0.2100 | 0.2580 | / | 0.4975 | / | 0.4781 | / |

Supplementary Table 6. Tear protein concentration of VEGF-A of the right (injured) eye measured at baseline (before injury), day 0, day 3 and day 7 after the injury for WT and Akita mice, respectively, n=8

| **VEGF-A**  **concentration [pg/ml]**  **Mean+SEM** | **Baseline** | **Day 0** | **p1** | **Day 3** | **p2** | **Day 7** | **p3** |
| --- | --- | --- | --- | --- | --- | --- | --- |
| **WT** | 2618 ± 818.2 | 1534 ± 1186 | 0.9178 | 3115 ± 1681 | 0.9909 | 1065 ± 521.8 | 0.7961 |
| **Akita** | 3423 ± 772.8 | 143.3 ± 59.82 | **0.0016** | 805.6 ± 573.1 | **0.0063** | 601.6 ± 243.8 | **0.0032** |
| **p4** | 0.4926 | 0.2623 | / | 0.1932 | / | 0.4158 | / |
