# Supplementary material for: The association between altered intestinal microbiome, impaired systemic and ocular surface immunity, and impaired wound healing response after corneal alkaline-chemical injury in diabetic mice
Source: Front Immunol. 2023 Jan 31;14:1063069. doi: 10.3389/fimmu.2023.1063069 (PMC9927643; doi:10.3389/fimmu.2023.1063069)
Supplement: Supplementary file 2 [file DataSheet_1.docx]

**Supplementary figures**


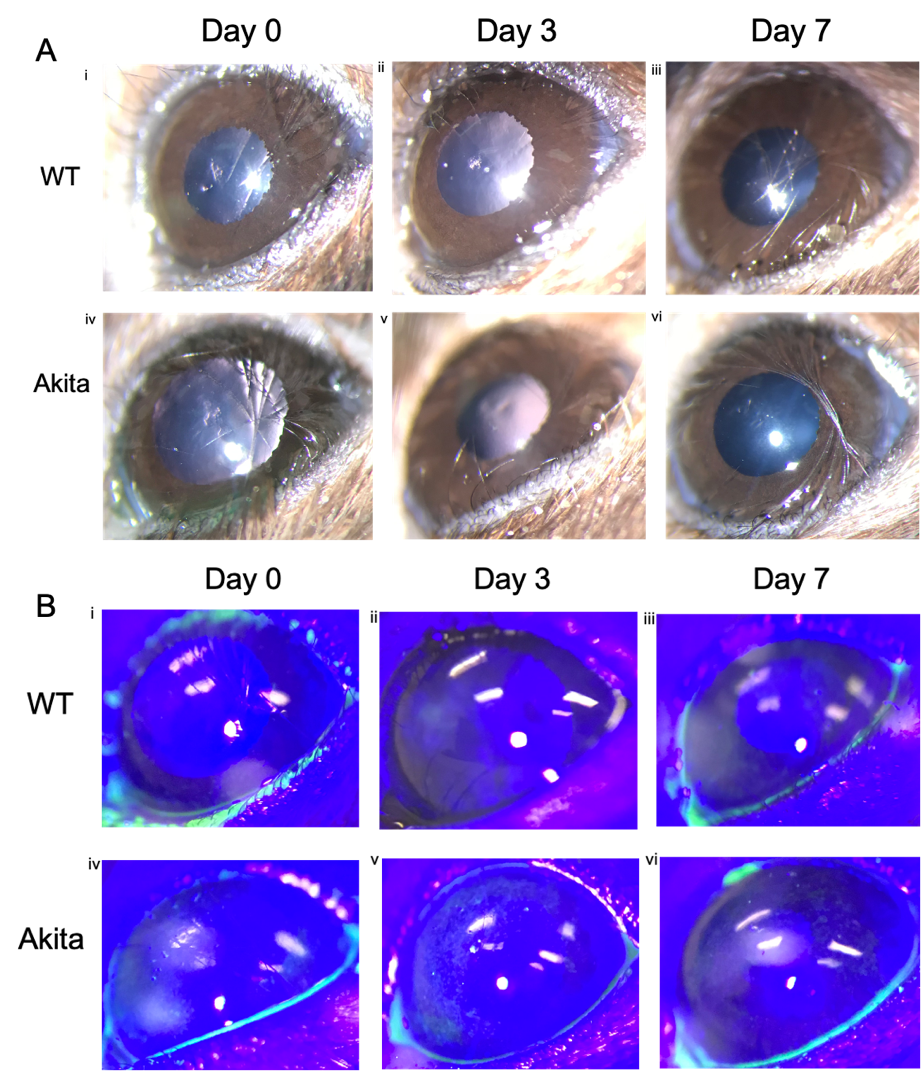


**Supplementary Figure 1. Slit lamp images for the left uninjured corneas serving as non-injury control group** A. Bright field image of the left (uninjured) cornea from WT (i, ii, iii) and Akita (iv, v, vi) mice at day 0, day 3 and day 7 after corneal alkaline burn injury conducted on the right eye of the mice. B. Fluorescein stain of the left (uninjured) cornea from WT (i, ii, iii and Akita (iv, v, vi) mice at day 0, day 3 and day 7 under cobalt blue light.
